# Association between venous thromboembolism and plasma levels of both soluble fibrin and plasminogen-activator inhibitor 1 in 170 patients undergoing total hip arthroplasty

**DOI:** 10.3109/17453674.2011.652886

**Published:** 2012-02-08

**Authors:** Yohei Yukizawa, Yutaka Inaba, Shin-ichiro Watanabe, Satoshi Yajima, Naomi Kobayashi, Takashi Ishida, Naoyuki Iwamoto, Hyonmin Choe, Tomoyuki Saito

**Affiliations:** ^1^Musculoskeletal Science, Yokohama City University Graduate School of Medicine; ^2^Department of Orthopaedic Surgery, Yokohama City University School of Medicine; ^3^Department of Clinical Laboratories, Yokohama City University Hospital, Yokohama, Japan

## Abstract

**Background and purpose:**

Markers of coagulation and fibrinolysis, such as soluble fibrin (SF), D-dimer, and plasminogen activator inhibitor 1 (PAI-1), have been developed in order to determine thrombotic tendency. We investigated whether these markers could be used to diagnose venous thromboembolism (VTE) in the early phase after primary total hip arthroplasty (THA).

**Methods:**

This prospective study involved 2 groups: an intermittent pneumatic compression (IPC) group (67 patients who underwent IPC only as prophylaxis for VTE) and a fondaparinux (FPX) group (103 patients who received IPC and FPX postoperatively). Plasma levels of SF and PAI-1 were measured on postoperative day 1. To diagnose postoperative VTE, multi-detector row computed tomography (MDCT) and duplex ultrasonography (US) were performed on postoperative day 7.

**Results:**

VTE was detected postoperatively in 17 cases in the IPC group (25%) and in 8 cases in the FPX group (6%). In the IPC group, plasma levels of SF and PAI-1 were higher in patients with VTE (p < 0.01) than in those without VTE. On the other hand, in the FPX group there were no differences in the levels of SF or PAI-1 measured before administration of FPX on postoperative day 1. The diagnostic criterion of an increase in SF or PAI-1 above the cutoff level (19.8 µg/mL and 53.5 ng/mL, respectively) provided a sensitivity of 100% and a specificity of 67% in the IPC group. In addition, when this criterion was applied to FPX patients, 7 of the 8 patients with VTE met the criterion, and there was a negative agreement rate of 48/49.

**Interpretation:**

Screening using the cutoff levels of SF and PAI-1 may be useful and shows high sensitivity in predicting postoperative VTE in the early phase after THA.

Randomized clinical trials have shown that the rate of deep vein thrombosis (DVT) after total hip arthroplasty (THA) in patients who do not receive thromboprophylaxis is 42–57% (Geerts et al. 2008). The American College of Chest Physicians (ACCP) recommends routine chemoprophylaxis using anticoagulant drugs after total joint arthroplasty of the hip or knee (Geerts et al. 2008). However, chemoprophylaxis generally involves the threat of postoperative bleeding, which must be balanced against the risk of thrombotic events.

In Japan, the frequency of postoperative venous thromboembolism (VTE) after THA when no anticoagulant prophylaxis is employed is 23–42% (Fujita et al. 2000, Fuji et al. 2008). The same emphasis has been put on VTE prophylaxis as in western countries since the first Japanese guidelines for VTE prophylaxis were prepared in 2004 (The first edition guidelines for prevention of venous thromboembolism, 2004). The latest guidelines limit chemoprophylaxis to a period of 14 days after surgery, and they also suggest intermittent pneumatic compression (IPC) without any administration of anticoagulants as one choice for postoperative prophylaxis because of its preventative effects (Sugano et al. 2009, Husted et al. 2010). Thus, there is a controversy among Japanese surgeons about what kind of thromboprophylaxis should be employed after major orthopedic surgery. Also in western countries, some authors have questioned whether routine chemoprophylaxis is necessary (Callaghan et al. 2005, Dorr et al. 2007). Thus, a useful screening method to determine which patients are at high risk of postoperative VTE should be of value.

Previously, tests for global screening of the coagulation system were considered to be unhelpful in the diagnosis of thrombotic events. However, recent biochemical studies of the coagulation and fibrinolysis systems have led to the availability of specific and sensitive tests that can detect thrombosis. At the same time, many different markers have been found to show increased expression in clinical disorders in which there is an imbalance between coagulation and fibrinolysis. We examined acute postoperative changes in (1) soluble fibrin (SF), a complex of fibrin monomer and fibrinogen derivatives; (2) thrombin-antithrombin complex (TAT), a marker of thrombin generation; (3) D-dimer, a proteolytic fragment resulting from degradation of a fibrin clot; and (4) plasminogen-activator inhibitor 1 (PAI-1), which is the main regulator of the fibrinolysis system. In addition, we evaluated the usefulness of assaying these markers as predictors of early VTE following THA.

## Patients and methods

170 patients with a mean age of 63 (38–85) years who were scheduled for THA between 2007 and 2010 were included. Patients were excluded if they had any of the following conditions: (1) a prior THA requiring revision; (2) a previous history of VTE; (3) a pre-existing malignant tumor; or (4) renal failure (estimated glomerular filtration rate < 50 mL·min-1·1.73 m-2). Before surgery, all patients were provided with a detailed explanation of the risks and alternatives to participation in the study, and all provided written informed consent. The study was approved by the Institutional Review Board of Yokohama City University (approval no., 01-10-2007-058).

### Prophylaxis for VTE

There were 2 patient groups: the IPC group (67 patients who underwent primary THA between 2007 and 2008) and the fondaparinux (FPX) group (103 patients who underwent THA between 2008 and 2010). At our institution, FPX has been used routinely since September 2008. IPC was performed on all patients in both groups during surgery (under anesthesia), and the patients were given unfractionated heparin (UFH) intravenously in a single dose of 20 IU/kg of body weight (Sharrock et al. 1999). IPC was maintained postoperatively. Patients usually started walking 1–2 days after surgery. The patients in the FPX group were also given 2.5 mg of FPX subcutaneously every day for 14 days, starting on postoperative day 1.

### Perioperative management

All patients were operated under general anesthesia. THA using a computed tomography-based navigation system was performed through a minimally-invasive anterolateral approach with the patient in the lateral decubitus position. During surgery, all patients received up to 1,500 mL of Ringer’s solution or hydroxyethyl starch. Fluid management after surgery until the next morning was routinely done with 1,000 mL of Ringer’s solution and 500 mL of maintenance fluid.

Postoperative mobilization followed a protocol supervised by experienced orthopedic physiotherapists. The patients were allowed discharge on postoperative day 14, the day this mobilization protocol was completed.

### Diagnostic methods for VTE

Duplex ultrasonography (US) was performed on all patients 28 days before surgery in order to detect any evidence of previous DVT of the lower limb. Absent or incomplete compressibility of the vein was the diagnostic criterion. For the determination of postoperative VTE, an angiography of the pulmonary artery and deep veins on the pelvis and the lower limbs was performed on all patients on postoperative day 7, by 64-slice multidetector row computed tomography (MDCT) using a nonionic contrast agent. The diagnostic criterion for VTE was the presence of a defect of intraluminal filling because of thrombosis in the pulmonary artery or deep vein. When the presence of VTE was suspected by MDCT, a duplex US was also employed in order to confirm the presence of DVT.

Following detection of the postoperative VTE, patients with VTE were administered UFH intravenously in order to maintain the activated partial thromboplastin time at 1.5–2.5 times that of the control value. Warfarin was administered at the same time, and the international normalized ratio was maintained at 2.0–2.5.

### Blood samples

Blood samples were obtained from peripheral veins under short fasting conditions early in the morning, on the preoperative day and on postoperative days 1, 3, 7, and 14. Plasma SF levels were measured using a latex photometric immunoassay (IATRO SF II; Mitsubishi Chemical Medience Corporation, Tokyo, Japan) with IF-43 monoclonal antibody raised against a urea-solubilized fibrin monomer. The normal upper limit for SF was < 7 µg/mL. PAI-1 was measured using a latex photometric immunoassay (LPIA-tPAI Test; Mitsubishi Chemical Medience Corporation) using a reference range of 10–50 ng/mL. Furthermore, preoperative levels of lipids such as triglycerides and total cholesterol were measured, and their association with PAI-1 was determined. Plasma D-dimer levels were also assayed with a latex photometric immunoassay (LPIA-ACE D-dimer; Mitsubishi Chemical Medience Corporation). The normal limit was < 0.7 µg/mL. TAT was measured by enzyme-linked immunosorbent assay (ELISA) with a reference range of 0.1–5.0 ng/mL (Enzygnost TATmicro; Siemens Healthcare Diagnostics Inc., Tokyo, Japan).

### Statistics

Statistical analyses were performed using SPSS II software. According to a Kolmogorov-Smirnov analysis, the coagulation and fibrinolysis variables showed a skewed distribution. Thus, these variables are presented as medians. The medians and interquartile ranges are plotted in the figures as a box-and-whisker plot. In the figures, the vertical bars (whiskers) represent the 5th and 95th percentiles and the horizontal bars in the boxes represent the medians. In addition, differences in the variables between patients with VTE and those without were examined statistically using Student’s t-test or a two-tailed Mann-Whitney U-test. Furthermore, the chi-square test and Fisher’s exact probabilities were used for the comparison between the observed and expected frequencies. The usefulness of the markers for the diagnosis of VTE was assessed by a receiver operating characteristic curve analysis. Values of p < 0.05 were considered statistically significant.

## Results

In the IPC group, postoperative VTE was detected in 17 of the 67 patients (25%), and the incidences of pulmonary embolism (PE) only, deep vein thrombosis (DVT) only, and both PE and DVT were 3, 12, and 2, respectively. In the FPX group, VTE was detected in 8 of the 103 patients (7%), and the incidences of PE only, DVT only, and both PE and DVT were 3, 5, and 0, respectively. The difference in the frequency of occurrence of VTE between the IPC and FPX groups was statistically significant (p < 0.01). All DVT occurred in the calf vein, and there were no cases of symptomatic DVT or PE. The distributions of age, sex, body mass index (BMI), duration of surgery, blood loss, and previous illness were similar in patients with and without VTE, and between the IPC and FPX groups (Table).

**Table:** 

	IPC group		FPX group		
	Patients with VTE	Patients without VTE	Patients with VTE	Patients without VTE	
Characteristics	n = 17	n = 50	n = 6	n = 97	p-value
Age, years **^a^**	68 (8)	62 (12)	58 (8)	61 (12)	0.1
Gender: male/female, n	3/14	17/33	6/0	21/76	0.5
Weight, kg **^a^**	58 (14)	58 (13)	59 (14)	58 (13)	0.9
Body mass index **^a^**	24 (6)	23 (5)	24 (5)	24 (5)	0.9
Primary hip disease, n					0.8
Osteoarthritis	15	36	4	79	
Rheumatoid arthritis	0	3	0	7	
ANFH	2	6	2	11	
PVNS	0	1	0	0	
Preoperative plasma levels of:					
Triglycerides, mg/dL **^a^**	92 (35)	108 (40)	99 (32)	98 (41)	0.7
Total cholesterol, mg/dL **^a^**	228 (44)	199 (31)	202 (26)	222 (36)	0.7
Operation length, min **^a^**	182 (62)	162 (36)	153 (39)	161 (37)	0.2
Blood loss, mL **^a^**	557 (193)	547 (217)	525 (137)	624 (265)	0.4

**^a^** Values are mean (SD).

IPC: intermittent pneumatic compression; FPX: fondaparinux sodium; VTE: venous thromboembolism; OA: osteoarthritis; RA: rheumatoid arthritis; ANFH: avascular necrosis of femoral head; PVNS: pigmented villonodular synovitis.

**Figure 1.**
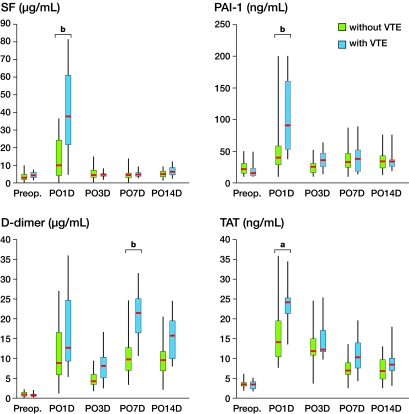
Changes in coagulation markers in patients who underwent intermittent pneumatic compression only. Plasma levels of soluble fibrin (SF), plasminogen activator inhibitor type 1 (PAI-1), D-dimer, and thrombin-antithrombin complex (TAT) in patients who underwent intermittent pneumatic compression alone were measured preoperatively and on postoperative days 1 (PO1D), 3 (PO3D), 7 (PO7D), and 14 (PO14D). The boxes represent the interquartile ranges. The perpendicular lines (whiskers) represent the fifth and ninety-fifth percentiles and the horizontal bars in the boxes indicate the median values. On the day after surgery, the plasma levels of SF, PAI-1, and TAT were found to be increased in the venous thromboembolism (VTE) group compared to the non-VTE group. The changes in the D-dimer levels showed bimodal peaks on postoperative days 1 and 7 in both groups. Statistically significant differences were observed in the D-dimer levels measured on postoperative day 7. **^a^** p < 0.05 and **^b^** p < 0.01.

**Figure 2.**
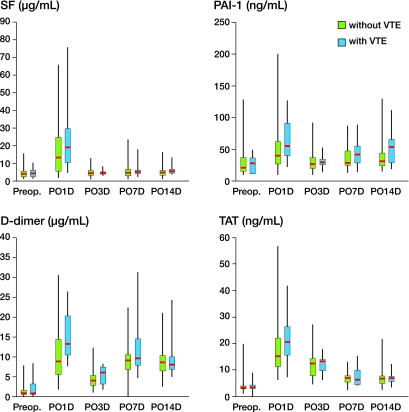
Changes in coagulation markers in patients who received subcutaneous injections of fondaparinux sodium. Plasma levels of soluble fibrin (SF), plasminogen activator inhibitor type 1 (PAI-1), D-dimer, and thrombin-antithrombin complex (TAT) in patients who received subcutaneous injections of fondaparinux sodium were measured preoperatively and on postoperative days 1 (PO1D), 3 (PO3D), 7 (PO7D), and 14 (PO14D). The boxes represent the interquartile ranges. The perpendicular lines represent the fifth and ninety-fifth percentiles and the horizontal bars in the boxes indicate the median values. The levels of SF, PAI-1, D-dimer, and TAT were similar in the patients in the fondaparinux group who had or did not have VTE.

In the IPC group, plasma SF levels on postoperative day 1 in patients with VTE were higher than those in patients without VTE (p < 0.01) (Figure 1), and the median values were 38 µg/mL and 10 µg/mL, respectively. Plasma SF levels rapidly decreased to reference range by postoperative day 3. On the other hand, in the FPX group SF levels were similar in patients with and without VTE (Figure 2).

In the IPC group, plasma PAI-1 levels on postoperative day 1 in patients with VTE were higher than those in patients without VTE (p < 0.01) (Figure 1). The median PAI-1 values on postoperative day 1 were 93 ng/mL for patients with VTE and 40 ng/mL for patients without VTE. In addition, in both the IPC and FPX groups plasma D-dimer levels showed bimodal peaks that were evident on postoperative days 1 and 7. In the IPC group, significant differences in the plasma D-dimer levels were not seen on postoperative day 1, but they were seen on postoperative day 7 (p < 0.01). The median D-dimer values on day 7 were 21 µg/mL for patients with VTE and 10 µg/mL for patients without VTE (Figures 1 and 2).

IPC patients with VTE also showed higher levels of TAT on postoperative day 1 (p < 0.05); the median values were 24 µg/mL for patients with VTE and 14 µg/mL for patients without VTE. On the other hand, statistically significant differences in TAT levels were not found in patients of the FPX group.

**Figure 3.**
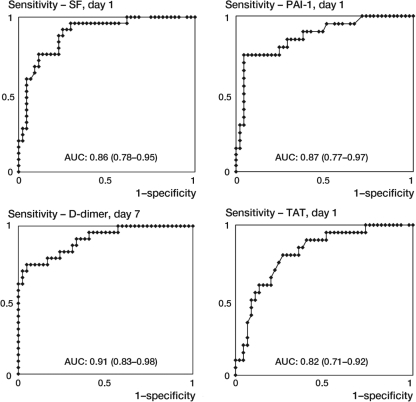
Receiver operating characteristic (ROC) curve analysis of the accuracy of the quantitative soluble fibrin (SF), plasminogen activator inhibitor type 1 (PAI-1), and thrombin-antithrombin complex (TAT) levels on postoperative day 1 and D-dimer levels on day 7. The area under the ROC curve (AUC) is given in each diagram with the 95% confidence interval in parentheses.

To evaluate the usefulness of the markers SF, PAI-1, and TAT that were detected in the IPC group on postoperative day 1, their cutoff levels with sensitivities and specificities were determined by receiver operating characteristic (ROC) analysis (Figure 3). The cutoff level of SF was determined to be 19.8 μg/mL, with a sensitivity of 88% and a specificity of 62%. The cutoff level of PAI-1 was 53.5 ng/mL, with a sensitivity of 78% and a specificity of 72%, and that of TAT was found to be 18.1 ng/mL with a sensitivity of 85% and a specificity of 66%. Next, using a multivariate logistic regression analysis, we found that SF and PAI-1 levels on postoperative day 1 had statistically the strongest association with thrombotic tendency. Furthermore, comparison of the area under the ROC curves showed that the measurements of SF and PAI-1 on postoperative day 1 had the best performance with respect to their discrimination ability (Figure 3). Also, there was no statistically significant correlation between the levels of PAI-1 and the levels of other coagulation markers.

**Figure 4.**
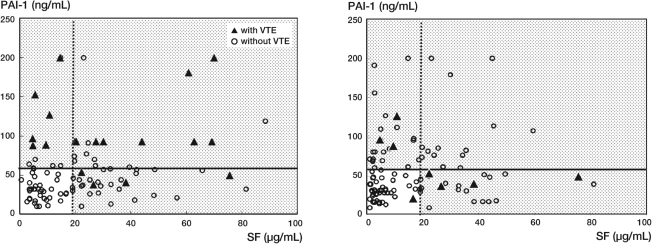
Discrimination of postoperative venous thromboembolism (VTE) using levels of soluble fibrin (SF) and plasminogen activator inhibitor type 1 (PAI-1). Increases in either SF or PAI-1 on postoperative day 1 above their cut-off levels provided 100% sensitivity and 67% specificity in predicting VTE when patients were not given fondaparinux sodium postoperatively (left panel). In addition, when this criterion was applied to patients who received subcutaneous injections of fondaparinux following surgery, 7 of the 8 patients with VTE met the criterion and a 98% (48/49) negative agreement rate was found (right panel).

Figure 4 shows scatter graphs of SF and PAI-1 levels, with 2 lines at each cutoff level. These lines divide the patients into 2 groups: those with higher marker levels and those with lower levels, and these divisions provided a sensitivity of 100%, a specificity of 67%, and a positive predictive value of 50%. In addition, when this criterion was applied to patients in the FPX group, 7 of the 8 with VTE met the criterion and there was a negative agreement rate of 98.0% (48/49).

## Discussion

VTE is a common complication after total hip or knee arthroplasty, but most VTE is generally asymptomatic. Due to the lack of symptoms, the condition goes mostly unnoticed and the patient is therefore left untreated. However, approximately 10–20% of crural thrombi, most of which are asymptomatic DVT, move to the proximal veins (Labropoulos et al. 2002). Proximal propagation of a thrombus can lead to free-floating status, resulting in critical complications after being released as a massive embolus (Baldridge et al. 1990). According to the PREVENT study (Ridker et al. 2003), the recurrence rate of VTE is approximately 7%, and long-term, low-intensity warfarin therapy reduces this risk by more than half. Thus, we suggest that all cases of VTE—including asymptomatic VTE—should be detected and treated with anticoagulants, and that an early and simple modality for screening of VTE should be established.

Highly invasive surgery has been shown to commonly result in a hypercoagulable state and increasing plasma levels of SF and TAT during the initial postoperative stage (Brueckner et al. 2003, Sudo et al. 2009). This is consistent with our present findings that plasma SF and TAT levels were elevated on postoperative day 1. Also, considering the substantial elevation of these markers in patients who developed VTE in the IPC group, we suggest that the onset of VTE is associated with a hypercoagulable state in the early phase after THA.

A high level of SF in clinical plasma samples has been recognized to be an indicator of ongoing intravascular coagulation processes (Hamano et al. 2005). SF expresses an acute intravascular fibrin formation as well because SF is one of circulating materials growing fibrin clots. Regarding the usefulness of SF for possible diagnosis of VTE, it was previously suggested that SF levels on the day after total hip or knee arthroplasty may be valuable for prediction of postoperative VTE (Sudo et al. 2009, Niimi et al. 2010).

Similarly to SF, TAT is considered to be associated with VTE following THA (Sudo et al. 2009). Formation of TAT is, however, only an indirect measure of an activated coagulation system (Brueckner et al. 2003), and its measurement is often influenced by the peripheral blood sampling techniques used under venous occlusion. Sensitivity and specificity of TAT measurement on the day after THA have been reported to be 73% and 27%, respectively (Cofrancesco et al. 1998). Thus, TAT has been found to be inferior to other markers as a predictor of VTE (Brueckner et al. 2003, Hamano et al. 2005).

PAI-1 is the principal inhibitor and critical regulator of plasminogen activator. Several clinical studies have found that plasma PAI-1 levels may be affected by lipid levels or vascular endothelial cell injury, and these factors may therefore increase the risk of thrombosis or embolism (Hamsten et al. 1987, Juhan-Vague et al. 1987). Furthermore, PAI-1 is produced at the site of inflammation following tissue injury. It has been reported that plasma PAI-1 levels are associated with surgical invasion, and that the increased levels of the fibrinolytic inhibitor that result from this may therefore be a major contributor to fibrinolytic shutdown (Kassis et al. 1992). In the present study, plasma PAI-1 levels were substantially elevated in IPC patients with VTE, despite the absence of any significant differences in other thrombotic risk factors such as lipid levels, BMI, or pre-existing diseases. Thus, we believe that changes in the fibrinolytic system may also be associated with the development of VTE.

Plasma D-dimer levels increase after the cleavage of formed thrombi by activated plasmin, and they have been suggested to be a useful marker for the diagnosis of VTE. The sensitivity and specificity for proximal DVT were 79% and 36%, respectively. In the present study, D-dimer measurements on postoperative day 7 were different in patients with and without VTE in the IPC group. However, at this stage, VTE is already evident, and we therefore suggest that D-dimer is not useful as an early predictor of this disorder.

An imbalance between coagulation and fibrinolysis contributes to excessive fibrin deposition in the vascular wall because both systems are composed of a complex cascade of molecules and closely influence each other (Aso 2007). Our results indicate that this imbalance can be detected with a combined assay involving SF as a coagulation marker and PAI-1 as a fibrinolytic marker. As shown in Figure 4, some of the patients with VTE had high levels of SF but low PAI-1 levels, whereas some had low SF levels and high PAI-1 levels. These data suggest that the onset of VTE after THA may be due to a hypercoagulable state, a highly regulated fibrinolytic state, or both.

We think that the combined measurement of SF and PAI-1 on postoperative day 1 is a useful screening method for patients who are at high risk of developing postoperative VTE. Our findings suggest that plasma levels of SF and PAI-1 on postoperative day 1 have the potential to provide an alternative chemoprophylaxis regimen for VTE after THA. However, the high sensitivity of this screening for prediction of VTE on day 7 was observed in patients receiving perioperative heparin and short-term IPC. It is necessary to investigate this further using different thromboprophylaxis methods and various imaging modalities. In addition, investigation of late VTE, which occurs 2–3 months after surgery, should be performed to confirm whether chemoprophylaxis is necessary for low-risk patients.

The levels of SF, PAI-1, TAT, and D-dimer in patients in the FPX group were similar between patients with and without VTE. This is because the use of FPX regulated the hypercoagulable state and the formation of thrombi, even in patients whose levels of coagulation markers were high in the early phase of surgery. Also, the reason why there were no differences in the levels of D-dimer between patients with and without VTE may be that FPX functioned as a treatment agent for already-developed VTE.

In both the IPC and FPX groups, VTE may have developed in some cases before the first measurements. Thus, delayed screening for VTE—i.e. on the day after the surgery—was not helpful, and screening should ideally be performed during or immediately after the surgery. Only 1 Japanese study has examined SF levels in the perioperative period following THA, and was unable to demonstrate the usefulness of these markers during or immediately after surgery because of a variety of factors. Although our measurements were delayed, our method is one of the fastest for screening of patients who are at high risk of developing VTE.

A 64-slice MDCT was employed as the imaging modality for the detection of postoperative VTE in this study, because with this technique one can simultaneously perform both pulmonary angiography and venography of the lower extremities. In addition, it is a less invasive technique than conventional ascending venography, requires considerably less time (approximately 5–8 min), and is technically simple (Lim et al. 2004). However, MDCT has several disadvantages, such as exposure of the patient to radiation, beam-hardening artifacts around the arthroplastic joint materials, and contrast-induced nephropathy. Concerning the accuracy of DVT detection since the advent of MDCT, several studies have shown that the diagnostic ability of indirect CT venography is comparable to US (Duwe et al. 2000, Loud et al. 2001, Lim et al. 2004), and both the sensitivity and the specificity in these studies ranged from 89–100%. US has recently become a widely accepted primary modality in the diagnosis of DVT because of its advantages, including high accuracy, painlessness, noninvasiveness, and reduced cost. Furthermore, US does not require radiation or contrast materials and there are no side effects. Thus, we employed duplex US for the preoperative screening of DVT and for postoperative confirmation of DVT suspected by MDCT.

In addition to imaging modalities, other factors may have affected the incidence of postoperative VTE, such as the method of anesthesia, duration of surgery, perioperative injection of heparin, immobilization, and duration of hospital stay (Husted et al. 2010, Brueckner et al. 2003). To minimize the influence of these factors on the occurrence of VTE, mobilization and rehabilitation directed by a physiotherapist were initiated within 24 hours of surgery. All patients underwent physiotherapy, and mobilization was frequently encouraged in free time during the hospital stay. Because of the limitation that the FPX injection can only be administered by nurses or doctors during the hospital stay and cannot be self-administered by patients at home, in Japan the duration of the hospital stay is longer than in other countries. The postoperative hypercoagulation state may change if epidural anesthesia is used (Brueckner et al. 2003) or if the duration of surgery is shortened. In the present study, a computed tomography-based navigation system was used to insert the joint implant accurately in all the patients. This system generally takes time to set and register. Furthermore, MIS techniques were used for all cases. It is possible that prolonged surgery may have resulted in the hypercoagulable state. Perioperative administration of heparin may also affect the incidence of postoperative VTE or the postoperative coagulation state, although the dose of heparin was considerably low.

In conclusion, patients undergoing primary THA are at a high risk of developing VTE, which is possibly induced by a hypercoagulable or regulated fibrinolytic state during the early postoperative phase. Plasma levels of SF and PAI-1 on day 1 after THA may be of value in providing an indication of the balance between coagulation and fibrinolysis, and in predicting VTE following THA. When high levels of SF or PAI-1 are observed on the day after surgery, there is a higher risk of postoperative VTE.
